# Diagnostic factors for recurrent pregnancy loss: an expanded workup

**DOI:** 10.1007/s00404-023-07001-z

**Published:** 2023-03-25

**Authors:** Carlo Ticconi, Elena Nicastri, Silvia D’Ippolito, Carlo Chiaramonte, Adalgisa Pietropolli, Giovanni Scambia, Nicoletta Di Simone

**Affiliations:** 1 Department of Surgical Sciences, Section of Gynecology and Obstetrics, University Tor Vergata, Rome, Italy; 2 U.O.C. di Ostetricia e Patologia Ostetrica, Dipartimento di Scienze della Salute della Donna, del Bambino e di Sanità Pubblica, Fondazione Policlinico Universitario A.Gemelli IRCCS, Rome, Italy; 3 Istituto di Clinica Ostetrica e Ginecologica, Università Cattolica del Sacro Cuore, Rome, Italy; 4 Department of Biomedicine and Prevention, University Tor Vergata, Viale Oxford 81, 00133 Rome, Italy; 5 U.O.C. di Ginecologia Oncologica, Dipartimento di Scienze della Salute della Donna, del Bambino e di Sanità Pubblica, Fondazione Policlinico Universitario A.Gemelli IRCCS, Rome, Italy; 6 Department of Biomedical Sciences, Humanitas University, Via Rita Levi Montalcini 4, 20072 Pieve Emanuele, Milan Italy; 7 Humanitas Clinical and Research Center, IRCCS, Via Manzoni 56, 20089 Rozzano, Milan Italy

**Keywords:** Recurrent pregnancy loss, Diagnostic factors, Risk factors, Pregnancy complications

## Abstract

**Purpose:**

There is limited information on the risk factors for recurrent pregnancy loss (RPL).

**Methods:**

In this study, a patient-based approach was used to investigate the possible involvement and relative relevance of a large number of diagnostic factors in 843 women with RPL who underwent an extensive diagnostic workup including 44 diagnostic factors divided into 7 major categories.

**Results:**

The rates of abnormalities found were: (1) genital infections: 11.74%; (2) uterine anatomic defects: 23.72%; (3) endocrine disorders: 29.42%; (4) thrombophilias: 62%; (5) autoimmune abnormalities: 39.2%; (6) parental karyotype abnormalities 2.25%; (7) clinical factors: 87.78%. Six hundred and fifty-nine out of eight hundred and forty-three women (78.17%) had more than one abnormality. The mean number of pregnancy losses increased by increasing the number of the abnormalities found (*r* = 0.86949, *P* < 0.02). The factors associated with the highest mean number of pregnancy losses were cervical isthmic incompetence, anti-beta-2-glycoprotein-1 antibodies, unicornuate uterus, anti-prothrombin A antibodies, protein C deficiency, and lupus anticoagulant. The majority of the considered abnormalities had similar, non-significant prevalence between women with 2 versus ≥ 3 pregnancy losses with the exception of age ≥ 35 years and MTHFR A1298C heterozygote mutation. No difference was found between women with primary and secondary RPL stratified according to the number of abnormalities detected (Chi-square: 8.55, *P* = 0.07). In these women, the only factors found to be present with statistically different rates were age ≥ 35 years, cigarette smoking, and genital infection by *Ureaplasma*.

**Conclusion:**

A patient-based diagnostic approach in women with RPL could be clinically useful and could represent a basis for future research.

**Supplementary Information:**

The online version contains supplementary material available at 10.1007/s00404-023-07001-z.

## What does this study add to the clinical work?

**Table:** 

This study investigated the potential usefulness and limits of a large panel of diagnostic factors for recurrent pregnancy loss.	

## Introduction

Recurrent pregnancy loss (RPL), defined as the spontaneous loss of two or more pregnancies [1] or the loss of two or more pregnancies before the 24th week of gestation [2], is a relevant complication of pregnancy affecting up to 5% of the couples of reproductive age [3]. It can actually be considered a true challenge for the contemporary obstetrics and reproductive medicine. Indeed, despite a considerable effort in basic and clinical research on RPL, still 40–50% of cases of RPL remain unexplained [4, 5]. This means that, even though several evidence-based risk factors for RPL have been identified [6, 7], many other potentially existent risk factors for RPL are still unknown, uncovered or poorly determined. However, the clear determination of the existence of a risk factor is difficult in women with RPL, due to great heterogeneity of these patients which often have substantial differences in age, number of previous pregnancy losses, type of RPL (primary–secondary), and lifestyles. Due to these reasons, a great number of patients and controls with the same clinical characterstics are required to clearly define a risk factor for RPL. Another major problem in this context is that the relative weight of each individual probable or possible risk factor in leading to RPL is not well determined, so that it can be difficult to accurately quantify the risk for future pregnancy loss to which a RPL patient is exposed. This is particularly relevant, also taking into account that RPL is believed to be a multifactorial condition [8–10], so that the copresence of multiple risk factors in a single woman could substantially change her future reproductive prognosis. Recently, our group developed a machine learning algorithm that stratifies patients into classes of risk using a supervised learning algorithm known as Support Vector Machines [11]. This method, in addition to the recently published International Guidelines [1, 2], might be a useful tool in the management of women with RPL. However, it needs the continuos support of a Medical Engineering Service to be applied to all patients which are referred everyday to the clinicians and, therefore, it can be used at present only in a research setting. Another potentially useful, and perhaps more simple approach, could be the assessment of the relative relevance of individual abnormalities found in the evaluation of women with RPL, by determining their association with the number of pregnancy losses in a population of women with RPL. The aim of the present study was to evaluate the potential clinical utility of this approach that, to our knowledge, has not been tested yet in women with RPL.

## Materials and methods

### Subjects and study design

The present retrospective study was carried out on 1,020 unselected women who consecutively attended as outpatients of the RPL Units of the Policlinico Tor Vergata University Hospital or of the Catholic University of the Sacred Hearth at the Policlinico Gemelli Hospital of Rome, Italy, from January 1, 2017 until March 30, 2021. All women were non-pregnant and their last pregnancy loss had occurred at least 2 months before their referral. They underwent the same standardized diagnostic protocol for RPL. This protocol, already reported in detail [12–14], included careful collection of general and obstetrical history, gynecologic examination, pelvic ultrasound, karyotype of both partners, hormonal profile, hysteroscopy, autoantibodies panel, metabolic evaluation of the female partner, screening for coagulation and thrombophilic disorders, cervical and vaginal swabs for the search of infective agents. The reproductive history of patients at referral was assessed by evaluating for each previous pregnancy the US findings and the trend over time of the serum hCG levels. Biochemical pregnancies, defined according to Kolte et al. [15], were included in the assessment since it has been shown that non-visualized pregnancy losses are relevant in the prognostic evaluation of women with RPL [16].

The present study was carried out in accordance with the Declaration of Helsinki, modified Tokyo 2004, and was approved by the Institutional Review Board (IRB) of Policlinico Tor Vergata University Hospital (protocol number: 42/19).

All the obtained data were used to build up a computerized database which was then used for the successive analyses. Any collected information was anonymized and de-identified prior to analysis.

Seven major categories of diagnostic factors were considered in the study: (1) genital infections; (2) uterine anatomic defects; (3) endocrine disorders; (4) thrombophilias; (5) autoimmune disorders; (6) parental karyotype abnormalities; (7) clinical factors: age ≥ 35, BMI ≥ 30, cigarette smoking; number of pregnancy losses ≥ 3.

The detail of the factors evaluated is the following:

1. Genital infections: *E. Coli. Streptococcus, Ureaplasma, Mycoplasma, Chlamydia, Genital Herpes Virus*
2. Uterine anatomical abnormalities: (a) congenital: bicorporeal uterus (Class U3), septate uterus (Class U2), hemi uterus (Class U4); (b) acquired: adenomyosis, uterine fibroids, uterine synechiae, cervical isthmic incompetence, uterine polyps
3. Endocrine disorders: diabetes mellitus, PCOS, thyroid disorders: hypothyroidism treated, anti-TPO (antibodies to thyroperoxidase), anti-TG (antibodies to thyroglobulin), high TSH (> 2.5 mIU/L)
4. Thrombophilic disorders: protein C deficiency, protein S deficiency, homozygous FVL mutation, heterozygous FVL mutation, high homocysteine, ATIII deficiency, homozygous G20210A mutation, heterozygous G20210A mutation, homozygous MTHFR C677T mutation, heterozygous MTHFR C677T mutation, homozygous MTHFR A1298C mutation, heterozygous MTHFR A1298C mutation
5. Autoimmune disorders: LAC (lupus anticoagulant), ACL (anti-cardiolipin antibodies), ANA (anti-nuclear antibodies), AMA (anti-mitochondria antibodies), ENA (extractable nuclear antigen antibodies), ASMA (antibodies to alpha-smooth muscle actin), Ds-DNA Ab (antibodies to double-strand DNA), β2-GP1 Ab (antibodies anti-beta-2-glycoprotein-1), Ab anti-prothrombin, Ab anti-annex V, Ab anti-gliadin, transglutaminase ab (antibodies anti-transglutaminase), Ab anti-endomysium
6. Parental karyotype abnormality
7. Clinical risk factors: age ≥ 35, BMI ≥ 30, cigarette smoking, pregnancy losses ≥ 3

The detail of the diagnostic factors investigated in the study, stratified according to the ESHRE Guidelines [2], is reported in Table 1. The factors not recommended by ESHRE have been referred to as “Other Factors” in the table.

**Table 1:** Detail of the diagnostic factors investigated in the study, stratified according to the ESHRE Guidelines [2]

Factors recommended by ESHRE guidelines	Other factors
Bicorporeal uterus	*E. Coli*
Septate uterus	*Streptococcus*
Hemi uterus	*Ureaplasma*, *Mycoplasma*
Thyroid disorders	*Chlamydia*
LAC (lupus anticoagulant)	*Genital herpes virus*
ACL (anti-cardiolipin antibodies)	Adenomyosis
ANA (anti-nuclear antibodies)	Uterine fibroids
β2-GP1 Ab (antibodies anti-beta-2-glycoprotein-1)	Uterine synechiae
BMI ≥ 30	Cervical isthmic incompetence
	Uterine polyps
	Diabetes mellitus
	PCOS
	Protein C deficiency
	Protein S deficiency
	Homozygous FVL mutation
	Heterozygous FVL mutation
	High homocysteine
	ATIII deficiency
	Homozygous G20210A mutation
	Heterozygous G20210A mutation
	Homozygous MTHFR A1298C mutation
	Heterozygous MTHFR A1298C mutation
	Homozygous MTHFR C677T mutation
	Heterozygous MTHFR C677T mutation
	AMA (anti-mitochondria antibodies)
	ENA (extractable nuclear antigen antibodies)
	ASMA (antibodies to alpha-smooth muscle actin)
	Ds-DNA Ab (antibodies to double-strand DNA)
	Ab anti-prothrombin
	Ab anti-annexin V
	Ab anti-gliadin
	Ab anti-transglutaminase
	Ab anti-endomysium
	Age ≥ 35
	Cigarette smoking
	Parental karyotype abnormality
	Pregnancy losses ≥ 3

The factors not recommended by ESHRE have been referred to as “Other Factors”

For each diagnostic factor, the patients were categorized as either normal or abnormal.

All the abnormal findings detected at the end of the diagnostic workup were included in one of the above major categories. Therefore, these categories included defined, probable or possible risk factors as well as other factors which resulted abnormal at the end of the diagnostic workup and whose role is still uncertain or unexplored. Then each abnormal factor/finding detected was matched with the mean number of pregnancy losses in the group of women in which it was present. To have an arbitrary measure of the potential impact of each individual abnormality detected, all the above abnormalities detected were stratified into two categories of risk/severity of RPL, constructed according to the number of pregnancy losses: low risk/severity—two pregnancy losses; high risk/severity—three or more pregnancy losses. Moreover, all the women included in the study were also evaluated by the above abnormalities according to the type of their RPL, primary or secondary.

### Definitions, diagnostic criteria, and tools

RPL was defined, according to the European Society of Human Reproduction and Embryology (ESHRE) [2], as two or more pregnancy losses before the 24th week of gestation. Primary RPL was defined as the absence of previous viable pregnancy beyond the 24th week of gestation; secondary RPL was defined as RPL occurring in women with at least one previous ongoing pregnancy beyond the 24th week of gestation.

Non-visualized pregnancy losses were defined according to Kolte et al. [16] and included women with pregnancy of unknown location (PUL) and women with biochemical pregnancy.

Uterine congenital abnormalities were defined according to the ESHRE/ESGE classification 2013 [17].

Adenomyosis was diagnosed according to the MUSA Group 2015 criteria [18].

To make the diagnosis of cervical isthmic incompetence, all the following criteria had to be fulfilled: (1) at least one pregnancy loss occurred in the second trimester before 20 weeks gestation without any other alternative explanation for the loss, (2) the histological absence of chorioamnionitis, (3) the documentation of a cervical length < 25 mm by transvaginal ultrasound.

- PCOS was diagnosed according to the Rotterdam revised consensus criteria [19].
- Diabetes was diagnosed according to the criteria of the American Diabetes Association [20, 21]
- Ultrasonography and hysteroscopy were used to diagnose uterine fibroids and endometrial polyps.
- Endometrial synechiae were diagnosed by hysteroscopy.
- The cutoff for normal TSH was serum level ≤ 2.5 mIU/L
- ANA were detected by indirect immunofluorescence. The threshold we adopted to consider women positive for ANA was 1:80 [12].

The diagnosis of anti-phospholipid syndrome (APLS) was established according to the criteria of Vomstein et al. [3].

More than 90% of the patients underwent genital, cervical, and vaginal swabs and blood tests in the laboratories of the two Centers involved in the present study.

### Inclusion and exclusion criteria

As a routine clinical practice for years in our Centers, special attention is given to carefully determine the characteristics of the previous pregnancy losses, in terms of both number and weeks of occurrence, to reach a reliable correlation between the anamnestic information and the clinical findings for each previous pregnancy. This is accomplished by examining, for each pregnancy loss, the concordance between the patient history with the results of the ultrasound examinations, of the beta-HCG determinations, as well as the findings of the clinical cards when hospital admittance occurred. This task is carried out independently by two members of the research group (one resident and one expert consultant) for each of the two Centers involved in the study. Only the cases in which the agreement by the two members was complete were included into the study. Conversely, all the cases in which a reliable concordance between history and biochemical, clinical and instrumental data was doubtful even for only one pregnancy were excluded from the study. All women who had RPL after IVF were excluded from the study.

Women who had incomplete data on all the diagnostic factors considered in the present study were excluded.

Women who had non-visualized pregnancy losses were included in the study.

### Statistical analysis

Kolmogorov–Smirnov test was used to analyze the distribution of the data. Data are presented as means ± SD or percentages, or odds ratios (OR) and 95% confidence intervals (CI) as appropriate. Statistical analysis was carried out by using Student’s *t* test and Chi-square test. Pearson’s coefficient was determined to analyze correlations. The distribution of each individual diagnostic factor between women with two and three RPLs and between women with primary and secondary RPL was evaluated by univariate analysis. Since the number of the diagnostic factors considered in the study was very high (*n* = 44), a reliable multivariate analysis could not be performed because the proper levels of significance would be 0.05/44 = 0.001363, calculated by applying the Bonferroni’s correction. To perform more than one hypothesis test similtaneously, the Holm–Bonferroni closed testing procedure was followed in which the single *P* values corresponding to the minimal hypotheses have been corrected according to their specific position in the ordinal scale of the respective levels of statistical significance.

The software used was the Statistical Software SPSS release 23. Significance was set at *P* < 0.05.

## Results

### Findings in the overall study population

Overall, 1020 women were initially included in the study. Of these, 177 (17.35%) were excluded in which an incomplete diagnostic workup was carried out or the documentation of the characteristics of the previous pregnancy losses was judged to be incomplete (Supplemental Fig. S1). Thus, the overall number of women included into the study was 843 patients in which 2373 pregnancy losses had occurred. The vast majority of miscarriages (90.91%, 2112 of 2373) in RPL women occurred before 12 weeks gestation. The major clinical characteristics of the study women are reported in Table 2. The rates of women with 2 (49.94%) and ≥ 3 (50.06%) pregnancy losses were almost equal; conversely, the women with primary RPL were higher than those with secondary RPL (63.10% vs. 36.89%, respectively; Chi-square test: 38.922, *P* < 0.00001). The age of women with secondary RPL (mean ± SD: 36.27 ± 4.88 years) was significantly higher than that of women with primary RPL (mean ± SD: 35.31 ± 5.80 years, Students’t *t* test: *P* = 0.014), while no differences were observed between the two subgroups with regard to: (1) BMI (kg/m^2^: 25.04 ± 5.75 in women with primary RPL and 25.49 ± 5.83 in women with secondary RPL, Students’t *t* test: *P* = 0.276), (2) mean pregnancy losses/woman (2.77 ± 1.06 and 2.88 ± 1.03 in women with primary and secondary RPL, respectively, Students’t *t* test: *P* = 0.142), (3) cigarette smoking: 191/532 (35.90%) in women with primary RPL versus 83/311 (26.68%), Chi-square test: 3.662, *P* = 0.056). The mean number of living children/woman in patients with secondary RPL was 1.28 ± 0.62. The stratification of the study women according to the number of pregnancy losses is shown in Table 3.

**Table 2:** Characteristics of study women

Subjects (*n*)	843
Age (years)	35.6 ± 5.50
BMI (kg/m^2^)	25.20 ± 5.70
Ethnicity (*n*) (%)	
Caucasian	756 (89.68%)
African	45 (5.33%)
Asian	27 (3.20%)
Hispanic	15 (1.77%)
Cigarette smoking (*n*) (%)	274 (32.50%)
Pregnancy losses (*n*)	2373
Mean pregnancy losses for woman	2.81 ± 1.05
Women with 2 pregnancy losses (*n*) (%)	421 (49.94%)
Women with ≥ 3 pregnancy losses (*n*) (%)	422 (50.06%)
Women with primary RPL (*n*) (%)	532 (63.10%)
Women with secondary RPL (*n*) (%)	311 (36.89%)
Mean gestational age of pregnancy loss (weeks)	8 ± 1.6

Data are expressed as *n*, mean ± SD or percentage

**Table 3:** Study women stratified by the number of pregnancy losses

Number of pregnancy losses	Number of women (%)
2	421 (49.94%)
3	250 (26.65%)
4	110 (13.05%)
5	42 (4.98%)
6	13 (1.54%)
7	4 (0.47%)
8	2 (0.23%)
9	1 (0.12%)

When the study women were classified according to the ESHRE Guidelines (2), 497/843 (58.95%) women resulted to have an established or probable cause of RPL and 346/843 (41.05%) women had unexplained RPL.

As expected, by taking into account the wide number of factors considered in the present study, the diagnostic workup resulted completely normal only in a very few women (23/843, corresponding to the 2.72% of the study population); in these women no, even minimal, abnormality was detected. In 820/843 women (97.28% of the study population), at least one abnormality was found. The rates of abnormalities found in the above categories of established and/or potential diagnostic factors considered were: (1) genital infections: 11.74%; (2) uterine anatomical abnormalities: 23.72%; (3) endocrine disorders: 29.42%; (4) thrombophilias: 62%; (5) autoimmune abnormalities: 39.2%; (6) parental karyotype abnormalities 2.25%; (7) clinical factors: 87.78%. Notably, at least one clinical abnormality was found in 740/843 women, corresponding to the highest percentage (87.78%). This was due to the high number of women who were ≥ 35 years (*n* = 514), who were smokers (*n* = 274), and who had ≥ 3 pregnancy losses (*n* = 422). Cervical incompetence (CI) was found in four patients, 0.47% of the total population studied. The gestational ages at which the pregnancy loss occurred in these women ranged 15–19 weeks.

The stratification of study women according to the number of abnormalities found revealed that the majority of women (659/843, corresponding to 78.17% of the overall population investigated) had more than one abnormality detected. Therefore, to investigate whether multiple abnormalities could have an effect on the number of pregnancy losses, the numbers of associations of the abnormalities detected were correlated with the mean numbers of pregnancy losses in the women in which they have been found. The mean number of pregnancy losses increased by increasing the number of the abnormalities found, with a statistically significant correlation (*r* = 0.86949, *P* < 0.02) (Table 4).

**Table 4:** Number of abnormalities found in study women with RPL (*n* = 843) in relation to the mean number of pregnancy losses

Number of abnormalities	Number of women (%)	Number of pregnancy losses (mean)
0	23 (2.72%)	46 (2)
1	161 (19.09%)	437 (2.71)
2	225 (26.69%)	629 (2.79)
3	238 (28.23%)	669 (2.81)
4	151 (17.91)	451 (2.98)
5	41 (4.86%)	41 (3.02)
6	4 (0.47%)	17 (4.25)

Pearson’s coefficient of correlation between number of abnormalities and mean number of pregnancy losses

*r* = 0.86949, *P* < 0.02

It is reasonable that at least several of all the detected abnormalities are actually unrelated to RPL. To better clarify this relevant aspect and find the potentially most relevant abnormalities, each individual factor included in the diagnostic workup was evaluated according to the mean number of pregnancy losses that occurred in the women in which it has been found to be abnormally present. The detailed results are reported in Table 5. Among the factors investigated, those associated with the highest mean number of pregnancy losses, in a decrescent order of magnitude, were cervical isthmic incompetence, anti-beta-2- glycoprotein-1 antibodies, unicornuate uterus, anti-Prothrombin A antibodies, protein C deficiency, and LAC. Nine women (1.06%) were diagnosed to have an APLS. The detailed results in women with ≥ 3 pregnancy losses are reported for comparison in Table 6.

**Table 5:** Detail of the association between the abnormalities found in women with RPL (*n* = 843), stratified by major diagnostic categories, and to the mean number of pregnancy losses

Categories of abnormalities	Number of abnormalities	Number of pregnancy losses	Mean number of pregnancy losses
Genital infections: 99 (11.74%) (99/843)			
*E. Coli*	23	70	3.04 ± 1.36
*Streptococcus*	31	95	3.06 ± 1.26
*Ureaplasma*, *Mycoplasma*	44	133	3.02 ± 1.13
*Chlamydia*	0	/	/
*Herpes virus*	19	49	2.57 ± 1.04
Uterine anatomical abnormalities: 200 (23.72%) (200/843)			
Bicorporeal uterus	4	10	2,.0 ± 0.50
Septate uterus	53	154	2.90 ± 0.99
Hemi uterus	4	13	3.25 ± 0.82
Adenomyosis	16	39	2.43 ± 0.78
Uterine fibroids	109	322	2.95 ± 1.32
Uterine synechiae	17	50	2.94 ± 0.99
Cervical isthmic incompetence	4	14	3.50 ± 0.50
Uterine polyps	42	113	2.69 ± 0.85
Endocrine disorders: 248 (29.42%) (248/843)			
Diabetes mellitus	26	79	3.03 ± 1.62
PCOS	21	60	2.85 ± 0.98
Thyroid disorders	223	639	2.86 ± 1.10
Thrombophilias: 522 (62%) (522/843)			
Protein C deficiency	20	63	3.15 ± 1.23
Protein S deficiency	14	42	3.00 ± 1.25
Homozygous FVL mutation	12	33	2.75 ± 0.92
Heterozygous FVL mutation	22	65	2.95 ± 1.18
High homocysteine	53	152	2.86 ± 1.16
ATIII deficiency	12	36	3.00 ± 0.91
Homozygous FII mutation	3	10	3.33 ± 1.24
Heterozygous FII mutation	26	71	2.73 ± 1.25
Homozygous MTHFR C677T mutation	130	364	2.80 ± 1.03
Heterozygous MTHFR C677T mutation	202	576	2.85 ± 1.09
Homozygous MTHFR A1298C mutation	68	181	2.66 ± 0.90
Heterozygous MTHFR A1298C mutation	172	508	2.95 ± 1.06
Autoimmune abnormalities: 330 (39.2%) (330/843)			
LAC	27	85	3.14 ± 1.14
ACL	30	92	3.06 ± 1.31
ANA	239	670	2.80 ± 1.10
AMA	8	25	3.12 ± 0.92
ENA	25	78	3.12 ± 1.27
ASMA	50	147	2.94 ± 1.13
Ab anti-DNA	10	24	2.40 ± 0.66
Ab anti-beta-2-glycoprotein-1	19	63	3.31 ± 1.21
Ab anti-prothrombin A	14	45	3.21 ± 1.01
Ab anti-annex V	19	49	2.57 ± 0.99
Ab anti-gliadin	7	21	3.00 ± 0.92
Ab anti-transglutaminase	6	15	2.50 ± 0.76
Ab anti-endomysium	5	13	2.60 ± 0.80
Parental karyotype abnormalities: 19 (2.25%) (19/843)	19	57	3.00 ± 1.02
Clinical factors: 740 (87.78%) (740/843)			
Age ≥ 35	514	1481	2.88 ± 1.06
BMI ≥ 30	158	438	2.77 ± 1.06
Cigarette smoking	274	768	2.80 ± 0.98
Pregnancy losses ≥ 3	422	1531	3.62 ± 0.94

**Table 6:** Detail of the association between the abnormalities found in women with with ≥ 3 miscarriages (*n* = 422)

Categories of abnormalities	Number of abnormalities	Number of pregnancy losses	Mean number of pregnancy losses
Infections: 48 (11.37%) (48/422)			
*E. Coli*	12	48	4.00 ± 1.29
*Streptococcus*	18	69	3.83 ± 1.16
*Ureaplasma*, *Mycoplasma*	26	97	3.73 ± 0.98
*Chlamydia*	0	/	/
*Herpes virus*	6	23	3.83 ± 1.06
Uterine anatomical abnormalities: 105 (24.88%) (105/422)			
Bicorporeal uterus	2	6	3.00 ± 0.00
Septate uterus	31	110	354 ± 0.83
Hemi uterus	3	11	3.66 ± 0.47
Adenomyosis	5	17	3.40 ± 0.80
Uterine fibroids	58	220	3.79 ± 1.34
Uterine synechiae	11	38	3.45 ± 0.89
Cervical isthmic incompetence	4	14	3.50 ± 0.50
Uterine polyps	20	69	3.45 ± 0.66
Endocrine disorders: 129 (30.56%) (129/422)			
Diabetes mellitus	12	51	4.25 ± 1.73
PCOS	11	40	3.63 ± 0.77
Thyroid disorders	119	431	3.62 ± 1.03
Thrombophilic disorders: 276 (65.40%) (276/422)			
Protein C deficiency	13	49	3.76 ± 1.12
Protein S deficiency	9	32	3.55 ± 1.25
Homozygous FVL mutation	6	21	3.50 ± 0.76
Heterozygous FVL mutation	11	43	3.90 ± 0.99
High homocysteine	27	100	3.70 ± 1.11
ATIII deficiency	8	28	3.50 ± 0.70
Homozygous FII mutation	2	8	4.00 ± 1.00
Heterozygous FII mutation	9	37	4.11 ± 1.28
Homozygous MTHFR C677T mutation	64	232	3.62 ± 0.91
Heterozygous MTHFR C677T mutation	107	386	3.60 ± 1.02
Homozygous MTHFR A1298C mutation	30	105	3.50 ± 0.76
Heterozygous MTHFR A1298C mutation	101	366	3.62 ± 0.91
Autoimmune disorders: 166 (39.33%) (166/422)			
LAC	17	65	3.82 ± 0.92
ACL	17	66	3.88 ± 1.23
ANA	115	422	3.66 ± 1.03
AMA	6	21	3.50 ± 0.76
ENA	14	56	4.00 ± 1.06
ASMA	27	101	3.74 ± 1.00
Ab anti-DNA	3	10	3.33 ± 0.47
Ab anti-glycoprotein-1	14	53	4.00 ± 1.41
Ab anti-prothrombin A	11	39	3.54 ± 0.89
Ab anti-annex V	7	25	3.57 ± 1.04
Ab anti-gliadin	4	15	3.75 ± 0.43
Ab anti-transglutaminase	2	7	3.50 ± 0.50
Ab anti-endomysium	2	7	3.50 ± 0.50
Parental karyotype abnormality: 12 (2.84%) (12/422)	12	43	3.58 ± 0.86
Clinical risk factors: 422 (100%) (422/422)			
Age ≥ 35	277	1007	3.63 ± 0.93
BMI ≥ 30	74	270	3.64 ± 0.99
Cigarette smoking	143	506	3.53 ± 0.85
Pregnancy losses ≥ 3	422	1531	3.62 ± 0.94

### Diagnostic factors in women with 2 versus ≥ 3 pregnancy losses

To verify whether differences could be detected in the prevalence of the abnormalities considered between women with 2 or ≥ 3 losses, all the abnormalities found were stratified into two major categories of risk (low and high risk) according to the mean number of pregnancy losses (2 or ≥ 3, respectively) that occurred in the women in which each specific abnormality has been found to be present. A threefold approach was used to investigate this issue. First, the number of abnormalities found in the diagnostic factors considered were compared in the two groups of women with 2 or ≥ 3 pregnancy losses. A significant difference was found (Chi-square: 35.49, *P* = 0.0000034), but it was mainly due to the fact that no abnormalities were present in any women with ≥ 3 losses (Supplemental Table S1). Then the abnormalities in the diagnostic factors considered were stratified by the mean numbers of pregnancy losses (2 or ≥ 3) in the study women. The abnormalities associated with the highest mean numbers of pregnancy losses were cervical isthmic incompetence, G20210A homozygote mutation, beta-2-glycoprotein-1 Ab, hemi uterus. The detailed results are reported in Table 7. Furthermore, the prevalence of each specific abnormal finding was compared in study women stratified by the number of pregnancy losses (2 or ≥ 3). The results are shown in Table 8. The majority of the considered abnormalities had similar, non-significant prevalence between the two groups of women. A significantly increased prevalence in women with ≥ 3 losses versus women with 2 losses was found only for age ≥ 35 years and MTHFR A1298C heterozygote mutation (Table 8).

**Table 7:** Stratification of all the abnormalities detected in 843 women with RPL, according to the number of pregnancy losses in women in which they have been found and divided in two major groups of risk—low (2 losses) and high risk (≥ 3 losses)—mean values are reported in a decreasing order of magnitude

Low risk—2 pregnancy losses (number of losses: mean ± SD)	High risk—≥ 3 pregnancy losses (number of losses: mean ± SD)
Heterozygous MTHFR A1298C mutation (2.95 ± 1.06)	Pregnancy losses ≥ 3 (3.62 ± 0.94)
Heterozygous FVL mutation (2.95 ± 1.18)	Cervical isthmic incompetence (3.50 ± 0.50)
Uterine fibroids (2.95 ± 1.32)	Homozygous G20210A mutation (3.33 ± 1.24)
ASMA (2.94 ± 1.13)	Ab anti-beta 2-glycoprotein-1 (3.31 ± 1.21)
Uterine synechiae (2.94 ± 0.99)	Hemi uterus (3.25 ± 0.82)
Septate uterus (2.90 ± 0.99)	Ab anti-prothrombin A (3.21 ± 1.01)
Age ≥ 35 (2.88 ± 1.06)	Protein C deficiency (3.15 ± 1.23)
High homocysteine (2.86 ± 1.16)	LAC (3.14 ± 1.14)
Thyroid disorders (2.86 ± 1.10)	ENA (3.12 ± 1.27)
PCOS (2.85 ± 0.98)	AMA (3.12 ± 0.92)
Heterozygous MTHFR C677T mutation (2.85 ± 1.09)	ACL (3.06 ± 1.31)
Cigarette smoke (2.80 ± 0.98)	*Streptococcus* (3.06 ± 1.26)
ANA (2.80 ± 1.10)	*E. Coli* (3.04 ± 1.36)
Homozygous MTHFR C677T mutation (2.80 ± 1.03)	Diabetes mellitus (3.03 ± 1.62)
BMI ≥ 30 (2.77 ± 1.06)	*Ureaplasma*/*Mycoplasma* (3.02 ± 1.13)
Homozygous FVL mutation (2.75 ± 0.92)	Parental karyotype abnormality (3.00)
Heterozygous G20210A mutation (2.73 ± 1.25)	ATIII deficiency (3.00 ± 0.91)
Uterine polyps (2.69 ± 0.85)	Ab anti-gliadin (3.00 ± 0.92)
Homozygous MTHFR A1298C mutation (2.66 ± 0.90)	Protein S deficiency (3.00 ± 1.25)
Ab anti-endomysium (2.60 ± 0.80)	
Ab anti-annex V (2.57 ± 0.99)	
*Herpes virus* (2.57 ± 1.04)	
Ab anti-transglutaminase (2.50 ± 0.76)	
Bicorporeal uterus (2.50 ± 0.50)	
Adenomyosis (2.43 ± 0.78)	
Ab anti-DNA (2.40 ± 0.66)

**Table 8:** Prevalence of specific abnormal findings in study women stratified by the number of pregnancy losses (2 or ≥ 3)

Specific abnormal finding	Women with ≥ 3 PL (*n* = 422) (%)	Women with 2 PL (*n* = 421) (%)	OR (95% CI) for ≥ 3 versus 2 losses	*P* value closed testing	
				Significance	Rank
*E. Coli*	12 (2.84%)	11 (2.61%)	1.09 (0.47–2.50)	*P* > 0.95, NS	39
*Streptococcus*	18 (4.26%)	13 (3.08%)	1.39 (0.67–2.89)	*P* > 0.95, NS	24
*Ureaplasma*	26 (6.16%)	18 (4.27%)	1.47 (0.79–2.72)	*P* > 0.95, NS	14
HSV	6 (1.42%)	13 (3.08%)	0.45 (0.17–1.20)	*P* = 0.55, NS	5
Bicorporeal uterus	2 (0.47%)	2 (0.47%)	0.99 (0.13–7.11)	*P* > 0.95, NS	44
Septate uterus	31 (7.34%)	22 (5.22%)	1.43 (0.81–2.52)	*P* > 0.95, NS	12
Hemi uterus	3 (0.71%)	1 (0.23%)	3.00 (0.31–29.0)	*P* > 0.95, NS	22
Adenomyosis	5 (1.18%)	11 (2.61%)	0.44 (0.15–1.29	*P* = 0.91, NS	7
Uterine fibroids	58 (13.74%)	51 (12.11%)	1.15 (0.77–1.72)	*P* > 0.95, NS	30
Uterine synechiae	11 (2.60%)	6 (1.42%)	1.85 (0.67–5.05)	*P* > 0.95, NS	15
Cervical isthmic incompetence	4 (0.94%)	0 (0%)	9.06 (0.48–168.89)	*P* > 0.95, NS	8
Uterine polyps	20 (4.73%)	22 (5.22%)	0.90 (0.48–1.67)	*P* > 0.95, NS	37
Diabetes mellitus	12 (2.84%)	14 (3.32%)	0.85 (0.38–1.86)	*P* > 0.95, NS	35
PCOS	11 (2.60%)	10 (2.37%)	1.10 (0.46–2.61)	*P* > 0.95, NS	38
Thyroid disorders	119 (28.19%)	104 (24.70%)	1.19 (0.88–1.62)	*P* > 0.95, NS	17
Protein C deficiency	13 (3.08%)	7 (1.66%)	1.87 (0.74–4.75)	*P* > 0.95, NS	11
Protein S deficiency	5 (1.18%)	9 (2.13%)	1.81 (0.60–5.45)	*P* > 0.95, NS	20
FVL homozygous mutation	6 (1.42%)	6 (1.42%)	0.99 (0.31–3.11)	*P* > 0.95, NS	43
FVL heterozygous mutation	11 (2.61%)	11 (2.60%)	0.99 (0.42–2.32)	*P* > 0.95, NS	42
Hyperhomocysteinemia	27 (6.39%)	26 (6.17%)	1.03 (0.59–1.81)	*P* > 0.95, NS	41
ATIII deficiency	4 (0.95%)	8 (1.89%)	2.01 (0.60–6.74)	*P* > 0.95, NS	19
G20210A homozygote mutation	2 (0.47%)	1 (0.23%)	2.00 (0.18–22.14)	*P* > 0.95, NS	33
G20210A heterozygous mutation	9 (2.13%)	17 (4.03%)	0.51 (0.22–1.17)	*P* = 0.66, NS	6
MTHFR C677T homozygous mutation	64 (15.16%)	66 (15.67%)	0.96 (0.66–1.39)	*P* > 0.95, NS	40
MTHFR C677T heterozygous mutation	107 (25.35%)	95 (22.56)	1.16 (0.84–1.60)	*P* > 0.95, NS	23
MTHFR A1298C homozygous mutation	30 (7.10%)	38 (9.02%)	0.77 (0.46–1.27)	*P* > 0.95, NS	21
MTHFR A1298C heterozygous mutation	101 (23.93%)	71 (16.86%)	1.55 (1.10–2.11)	***P* = 0.0011**	1
LAC	17 (4.02%)	10 (2.37%)	1.72 (0.78–3.81)	*P* > 0.95, NS	10
ACL	17 (4.02%)	13 (3.08%)	1.31 (0.63–2.74)	*P* > 0.95, NS	28
ANA	115 (27.25%)	124 (29.45%)	0.89 (0.66–1.21)	*P* > 0.95, NS	29
AMA	6 (1.42%)	2 (0.47%)	3.02 (0.60–15.05)	*P* > 0.95, NS	9
ENA	14 (3.31%)	11 (2.61%)	1.27 (0.57–2.85)	*P* > 0.95, NS	31
ASMA	27 (6.39%)	23 (5.33%)	1.18 (0.66–2.09)	*P* > 0.95, NS	32
Ds-DNA Ab	3 (0.71%)	7 (1.66%)	0.42 (0.10–1.64)	*P* > 0.95, NS	13
β2-GP1 Ab	14 (3.31%)	5 (1.18%)	2.85 (1.01–7.99)	*P* = 0.184, NS	4
Ab anti-prothrombin	11 (2.60%)	3 (0.71%)	3.72 (1.03–13.46)	*P* = 0.132, NS	3
Ab anti-annex V	7 (1.65%)	12 (2.85%)	0.57 (0.22–1.47)	*P* > 0.95, NS	16
Ab anti-gliadin	4 (0.94%)	3 (0.71%)	1.33 (0.29–5.99)	*P* > 0.95, NS	36
Transgutaminase-Ab	2 (0.47%)	4 (0.95%)	0.49 (0.09–2.72)	*P* > 0.95, NS	27
Ab anti-endomysium	2 (0.47%)	3 (0.71%)	0.66 (0.11–3.99)	*P* > 0.95, NS	34
Parental karyotype abnormality	12 (2.84%)	7 (1.66%)	1.73 (0.67–4.44)	*P* > 0.95, NS	18
Maternal age ≥ 35 y	277 (65.63%)	237 (56.29%)	1.48 (1.12–1.95)	***P* = 0.011**	2
BMI ≥ 30 kg/m^2^	74 (17.53%)	84 (19.95%)	0.85 (0.60–1.20)	*P* > 0.95, NS	25
Cigarette smoke	143 (33.88%)	131 (31.11%)	1.13 (0.85–1.51)	*P* > 0.95, NS	26

Statistically significant comparisons are indicated in bold characters

*PL* pregnancy losses, *NS* not significant

### Diagnostic factors in women with primary versus secondary RPL

To evaluate the impact of the abnormalities investigated in the diagnostic workup on the type of RPL, the study women with ≥ 3 RPL were stratified into primary and secondary groups. No significant differenes were found in the rates of pregnancy losses between the two groups of women (OR = 1.041, 95% CI 0.885–1.225, *P* = 0.62). No overall significant difference was found between women with primary and secondary when they were stratified according to the number of abnormalities detected (Chi-square: 8.55, *P* = 0.07). The only significant difference was found when the women were stratified by the presence of only one diagnostic factor (Supplemental Table S2).

In women with primary and secondary RPL with ≥ 3 pregnancy losses, the impact of the individual factors included in the diagnostic workup was also evaluated according to the mean number of pregnancy losses that occurred in women in which it has been found to be abnormally present. The abnormalities associated with the highest mean number of pregnancy losses in women with primary RPL were Ab anti-prothrombin A, homozygous G20210A mutation, ACL, protein S and protein C deficiency. These abnormalities were different from those found in women with secondary RPL, that were cervical isthmic incompetence, Ab anti-glycoprotein-1, ENA, Ab anti-endomysium, hemi uterus, diabetes mellitus. The detail of all the abnormalities detected is reported in Table 9.

**Table 9:** Detail of the abnormalities detected in women with RPL with mean number of pregnancy losses ≥ 3 stratified by the type of RPL—primary versus secondary

Primary RPL (mean number of pregnancy losses)	Secondary RPL (mean number of pregnancy losses)
Ab anti-prothrombin A (3.66 ± 1.24)	Cervical isthmic incompetence (4 ± 0)
Homozygous FII mutation (3.5 ± 1.50)	Ab anti-glycoprotein-1 (3.77 ± 1.31)
ACL (3.21 ± 1.54)	ENA (3.62 ± 1.11)
Protein S deficiency (3.18 ± 1.33)	Ab anti-endomysium (3.5 ± 0.5)
Protein C deficiency (3.16 ± 1.40)	Hemi uterus (3.5 ± 0.5)
LAC (3.16 ± 1.25)	Diabetes mellitus (3.5 ± 2.34)
*Streptococcus* (3.14 ± 1.31)	*E. coli* (3.37 ± 1.21)
*Ureaplasma*, *Mycoplasma* (3.02 ± 1.18)	AMA (3.33 ± 0.47)
Cervical isthmic incompetence (3 ± 0)	Ab anti-annexin V (3.33 ± 1.37)
ASMA (3.06 ± 1.31)	ATIII deficiency (3.25 ± 0.82)
Hemi uterus (3 ± 1.0)	Heterozygous MTHFR A1298C mutation (3.14 ± 1.05)
AMA (3 ± 1.09)	LAC (3.11 ± 0.87)
Ab anti-gliadin (3 ± 0.92)	Protein C deficiency (3.12 ± 0.92)
Parental karyotype abnormality (3 ± 1.24)	Septate uterus (3.08 ± 1.11)
	Heterozygous MTHFR C677T mutation (3.04 ± 1.24)
	*Ureaplasma*, *Mycoplasma* (3 ± 0.86)
	Uterine fibroids (3 ± 1.32)
	Uterine synechiae (3 ± 0.63)
	Homozygous FVL mutation (3 ± 0)
	Homozygous FII mutation (3 ± 0)
	Ab anti-transglutaminase (3 ± 1.00)
	Parental karyotype abnormality (3 ± 0.63)

Mean values of pregnancy losses are reported in a decreasing order of magnitude

The prevalence of specific abnormal findings in all study women, including patients with two previous losses, stratified by primary and secondary RPL, is shown in Table 10. The only factors found to be present with statistically different rates between women with primary and secondary RPL were maternal age ≥ 35 years, cigarette smoking, and genital infection by *Ureaplasma*. PCOS prevalence was higher in women with primary RPL, however, without reaching statistical significance (Table 10).

**Table 10:** Prevalence of specific abnormal findings in study women stratified by primary and secondary RPL

Specific abnormal finding	Primary (*n* = 532) (%)	Secondary (*n* = 311) (%)	OR (95% CI)	*P* value closed testing	
				Significance	Rank
*E. Coli*	15 (2.81%)	8 (2.57%)	1.09 (0.46–2.62)	*P* > 0.95, NS	39
*Streptococcus*	21 (3.94%)	10 (3.21%)	1.23 (0.57–2.66)	*P* > 0.95, NS	27
*Ureaplasma*	36 (6.76%)	8 (2.57%)	2.74 (1.26–5.99)	***P* = 0.033**	3
*HSV*	16 (3.0%)	3 0.96%)	3.18 (0.92–11.01)	*P* = 0.36, NS	6
Bicorporeal uterus	4 (0.75%)	0 (0%)	5.30 (0.28–98.86)	*P* > 0.95, NS	16
Septate uterus	41 (7.70%)	12 (3.8%)	2.08 (1.07–4.02)	*P* = 0.145, NS	5
Hemi uterus	2 (0.37%)	2 (0.64%)	0.58 (0.08–4.15)	*P* > 0.95, NS	28–29
Adenomyosis	11 (2.06%)	5 (1.60%)	1.29 (0.44–3.75)	*P* > 0.95, NS	33
Uterine fibroids	75 (14.09%)	34 (10.93%)	1.33 (0.86–2.05)	*P* > 0.95, NS	12
Uterine synechiae	12 (2.25%)	5 (1.60%)	1.41 (0.49–4.04)	*P* > 0.95, NS	26
Cervical isthmic incompetence	2 (0.37%)	2 (0.64%)	0.58 (0.08–4.15)	*P* > 0.95, NS	28–29
Uterine polyps	32 (6.01%)	10 (3.21%)	1.92 (0.93–3.97)	*P* > 0.95, NS	8
Diabetes mellitus	18 (3.38%)	8 (2.57%)	1.32 (0.56–3.08)	*P* > 0.95, NS	24–25
PCOS	20 (3.75%)	1 (0.32%)	12.10 (1.61–90.67)	*P* = 0.06, ﻿NS	4
Thyroid disorders	149 (28.0%)	74 (23.79%)	1.24 (0.90–1.72)	*P* > 0.95, NS	11
Protein C deficiency	12 (2.25%)	8 (2.57%)	0.87 (0.35–2.16)	*P* > 0.95, NS	37
Protein S deficiency	11 (2.06%)	3 (0.96%)	2.16 (0.60–7.83)	*P* > 0.95, NS	14
FVL homozygous mutation	11 (2.06%)	1 (0.32%)	6.54 (0.84–50.94)	*P* > 0.95, NS	7
FVL heterozygous mutation	16 (3.0%)	6 (1.92%)	1.57 (0.61–4.07)	*P* > 0.95, NS	20
Hyperhomocysteinemia	35 (6.57%)	18 (5.78%)	1.14 (0.63–2.06)	*P* > 0.95, NS	34
ATIII deficiency	8 (1.50%)	4 (1.28%)	1.17 (0.34–3.92)	*P* > 0.95, NS	38
G20210A homozygote mutation	2 (0.37%)	1 (0.32%)	1.16 (0.10–12.95)	*P* > 0.95, NS	42
G20210A heterozygous mutation	18 (3.38%)	8 (2.57%)	1.32 (0.56–3.08)	*P* > 0.95, NS	24–25
MTHFR C677T homozygous mutation	87 (16.35%)	43 13.82%)	1.21 (0.82–1.80)	*P* > 0.95, NS	17
MTHFR C677T heterozygous mutation	130 (24.43%)	72 (23.15%)	1.07 (0.77–1.49)	*P* > 0.95, NS	35
MTHFR A1298C homozygous mutation	41 (7.70%)	27 (8.68%)	0.87 (0.52–11.45)	*P* > 0.95, NS	31
MTHFR A1298C heterozygous mutation	115 (21.61%)	57 (18.32%)	1.22 (0.86–1.75)	*P* > 0.95, NS	15
LAC	18 (3.38%)	9 (2.89%)	1.17 (0.52–2.64)	*P* > 0.95, NS	36
ACL	19 (3.57%)	11 (3.53%)	1.01 (0.47–2.15)	*P* > 0.95, NS	44
ANA	143 (26.87%)	96 (30.86%)	0.82 (0.60–1.14)	*P* > 0.95, NS	13
AMA	5 (0.93%)	3 (0.96%)	0.97 (0.23–4.10)	*P* > 0.95, NS	43
ENA	17 (3.19%)	8 (2.57%)	1.25 (0.53–2.93)	*P* > 0.95, NS	30
ASMA	29 (5.45%)	21 (9.96%)	0.79 (0.44–1.42)	*P* > 0.95, NS	23
Ds-DNA Ab	5 (0.93%)	5 (1.60%)	0.58 (0.16–2.02)	*P* > 0.95, NS	22
β2-GP1 Ab	10 (1.87%)	9 (2.89%)	0.64 (0.25–1.59)	*P* > 0.95, NS	19
Ab anti-prothrombin	6 (1.12%)	8 (2.57%)	0.43 (0.14–1.25)	*P* > 0.95, NS	9
Ab anti-annexin V	13 (2.44%)	6 (1.92%)	1.27 (0.47–3.38)	*P* > 0.95, NS	32
Ab anti-gliadin	7 (1.31%)	0 (0%)	8.89 (0.50–156.22)	*P* > 0.95, NS	10
Transgutaminase-Ab	4 (0.75%)	2 (0.64%)	1.17 (0.21–6.42)	*P* > 0.95, NS	40
Ab anti-endomysium	3 (0.56%)	2 (0.64%)	0.87 (0.14–5.27)	*P* > 0.95, NS	41
Parental karyotype abnormality	14 (2.63%)	5 (1.60%)	1.65 (0.59–4.63)	*P* > 0.95, NS	18
Maternal age ≥ 35 y	306 (57.51%)	208 (66.88%)	0.67 (0.50–0.89)	***P* = 0.0146**	**2**
BMI ≥ 30 kg/m^2^	95 (17.21%)	63 (20.25%)	0.85 (0.60–1.21)	*P* > 0.95, NS	21
Cigarette smoke	191 (35.90%)	83 (26.68%)	1.53 (1.13–2.09)	***P* = 0.0060**	**1**

Statistically significant comparisons are indicated in bold characters

*NS* not significant

## Discussion

The clinical research on RPL can be problematic for many aspects. This is due, in addition to several potential methodological pitfalls [22], to many reasons including the following: (a) the relative rarity of the condition can limit the size of the subjects studied; (b) the discrepancies in the definition of RPL make comparisons among the studies difficult [23]; (c) the heterogeneity of the women with RPL prevents a proper stratification of the subjects with specific conditions; (d) a substantial number of cases is currently classified as unexplained due to the still largely incomplete knowledge of the mechanisms underlying RPL; this limits the possibility to offer a reliable prognostic perspective and a non-empirical treatment to many women with RPL.

In this context, the evaluation of risk factors for RPL is particularly difficult. In fact, at present, only limited risk factors for RPL have been identified with fair reliability [6, 7, 23], so that a cause for RPL can be identified in fewer than 50% of couples [5].

The best way to thoroughly establish whether a suspected or potential factor is actually present implies that the factor of interest has a biological and/or clinical plausibility, is present in women with RPL, and is absent or less frequently present in women without RPL. This would require the recruitment of an extremely large number of women with RPL and an extremely large number of control women without RPL to be compared for the coexistence of other multiple established, potential or possible risk factors. However, this type of study is hard to be carried out, not only for the numbers of subjects to be included, but also for the enormous costs of testing many factors, substances, and conditions in otherwise healthy women, as well as for the relevant ethical implications. Moreover, the relative relevance of each individual risk factor detected remains largely undetermined. On the basis of the above considerations, a patient-based approach in the evaluation of the potential relevance of the risk factors or abnormalities detected in women with RPL could be useful. We decided to investigate whether such an approach could offer potentially useful clinical information since we had the possibility to perform, in a relatively large cohort of women with RPL, an extensive diagnostic workup which included the investigations recommended by current guidelines with several additional diagnostic factors currently considered of low, limited or uncertain relevance in determining RPL. Many, if not most, of these factors are out of the scope of the current guidelines and their clinical significance, if any, is still uncertain or undetermined; however, they are often required, even though in a scattered manner, in the everyday clinical practice in patients with RPL.

The results of the present research showed that only in a very small number of women, corresponding to 2.72% of the women included in the study, any abnormality was detected. This rate is very different from the 41.3% rate of unexplained RPL obtained by applying the ESHRE Guidelines to our study population [2]. This finding was expected, due to the large panel of items evaluated, and does not mean that the women in this very restricted group actually have a “truly unexplained” RPL. Rather, it indicates that several abnormalities detected during the diagnostic workup have been only incidentally found and are very likely to be unrelated to RPL. However, we found a significant correlation between the number of abnormal diagnostic factors considered in the study and the number of pregnancy losses in the study population of women with RPL (Table 4), showing a possible additive effect. This finding further supports the concept that RPL is actually a multifactorial condition [9]. Notably, the presence of at least one clinical abnormality was found in more than 87% of the study women, suggesting the relevance of the thorough collection of major clinical data in the overall evaluation of women with RPL. The high rate of clinical factors detected in the studied women can be explained by the following considerations: (1) we decided to adopt maternal age ≥ 35 years as a threshold of increased risk on the basis of observations recognized by the ESHRE [2, 25, 26], even though recent evidence indicates that the maternal age ≥ 40 years could have a more definite impact on the prognosis of subsequent pregnancies [27]; (2) we considered the number of pregnancy losses ≥ 3 as expression of increased severity of the condition [27, 28]; (3) the inclusion of cigarette smoking as a definite clinical risk factor is in accordance with the findings of a recent multivariable prediction model [29].

There is limited knowledge on the strength of specific factors in determining RPL. In the present study, we approached this issue by calculating the mean number of pregnancy losses in the women in which each single diagnostic factor was present and stratified the diagnostic factors considered by two major risk categories (< 3 and ≥ 3 pregnancy losses). Interestingly, nearly all the evidence-based diagnostic factors for RPL were included in the high-risk group: cervical incompetence, beta-2-glycoprotein IgM and IgG antibodies, unicornuate uterus, LAC, ACL, parental karyotype abnormalities (Table 4). The observation that several genital infections, namely by *Steptococcus*, *E. Coli*, *Ureaplasma/Mycoplasma*, fall into the high-risk group does not absolutely imply that these microbial agents are, at least to some extent, responsible for RPL in the women in which they have been found even if recent experimental evidence suggests that chronic endometritis, as consequence of genital infection, is becoming an established causative factor of RPL [30–33]. At present, we cannot establish whether the above microbial agents are actually involved in RPL because we did not perform endometrial biopsy or cultures in our studied women, since these procedure were included only recently in our diagnostic workup. Nonetheless, we believe that the potential involvement of genital infections by selected microbial agents deserves consideration in future studies on RPL. This concept is supported by recent evidence showing that differences exist in the uterine microbiota of women with RPL and RIF compared with healthy women [34]. ANA were detected in nearly 30% of our study population (Table 5). Their role in RPL is still incompletely determined. However, several recent meta-analyses [35–37] showed that ANA can exert a detrimental effect on several reproductive processes, including RPL. In our study, a very low number of women (*n* = 9) were diagnosed to have APLS. This low rate (1.06%) is in accordance with that found (1.44%) in a study recently published in which, additionally, it has been shown that similar rates of inherited thrombophilia have been found between RPL and control women [38].

In a large retrospective study, Jaslow et al. [39] investigated whether differences could be found in the prevalence of multiple diagnostic factors (established and probable) between women with two or three or more pregnancy losses. In this pioneering study, they found no differences irrespective of the number of previous pregnancy losses. As a part of the present study, we investigated the same issue, with a different statistical approach and a larger panel of diagnostic factors considered. The results of our study, while taking into account the above differences, substantially confirm that no differences could be detected (Table 8). The only two exceptions are the maternal age > 35 years and the MTHFR A1298C heterozygous mutation; however, the respective O.Rs. (1.48 and 1.55) were actually low. While there is consensus on the impact of advanced maternal age on RPL, the role of MTHFR A1298C mutation is considered not relevnt [2]. However, a recent meta-analysis including more than 14.000 subjects showed a significant association of the MTHFR A1298C polymorphism with RPL in the Caucasian populations [40].

There is little information about differences in risk factors in women with primary and secondary RPL. As a final part of the present study, we investigated whether differences between women with primary and secondary RPL could be found in the prevalence of the diagnostic factors considered. As a general consideration, no substantial differences were found between women with primary and secondary RPL. As far as we know, these findings are novel. The major significant difference was in the cigarette smoking, followed by maternal age and genital *Ureaplasma* infection, although once again the O.Rs. were rather low (Table 10).

Before drawing firm conclusions, the strenghts and limitations of the study need to be discussed. A major strenght of our study is the large number of diagnostic factors investigated at the same time in the same patient in a relatively large population of selected women with RPL. The correlation of each individual diagnostic factor with the mean of pregnancy losses in women in which it was found can suggest the relative relevance of the studied factor as a determinant of RPL. The detailed stratification of the study women, required to properly investigate women with RPL, which are very heterogenoeus, represents another strength of the study, whose results further support the concepts that RPL is a multifactorial condition and that little differences occur between women with two or more pregnancy losses as well as beteween women with primary and secondary RPL.

A major limitation of our study is its retrospective nature. Moreover, the study is limited to women with RPL without normal control patients. This is due to the huge costs that would be required to carry out a controlled, prospective study taking into account all the diagnostic factors we considered also in a control population; in addition to the cost issue, relevant ethical questions could be raised in performing such enlarged diagnostic workup in an otherwise healthy population. This is the same problem encountered in other well-conducted studies in which reference populations were used as controls [39]. We decided to not use this approach, focusing only on women with RPL. Of course, and also for the above reasons, we could not establish with certainty the involvement of all the diagnostic factors investigated in RPL. Therefore, the clinical significance of the approach presented in our study is still uncertain. Probably at least several factors are not involved; however, we could obtain an indication on the possible impact of the above factors by matching their presence with the mean number of pregnancy losses in our patients. The results obtained suggest that such an approach could be of some value and could represent a basis for future investigations on RPL. Further study is required to confirm the clinical value of a patient-based approach in the diagnostic workup of women with RPL.

## Supplementary Information

Below is the link to the electronic supplementary material.

Supplementary file 1 (PPTX 36 KB)

Supplementary file 2 (DOCX 12 KB)

Supplementary file 3 (PDF 294 KB)

Supplementary file 4 (DOCX 15 KB)

Supplementary file 5 (DOCX 15 KB)

## Data Availability

Data are available on reasonable request.
