# Supplementary material for: Diagnostic factors for recurrent pregnancy loss: an expanded workup
Source: Arch Gynecol Obstet. 2023 Mar 25;308(1):127–42. doi: 10.1007/s00404-023-07001-z (PMC10191960; doi:10.1007/s00404-023-07001-z)
Supplement: Supplementary file 1 — (PPTX 36 KB) [file 404_2023_7001_MOESM1_ESM.pptx]

## Slide 1
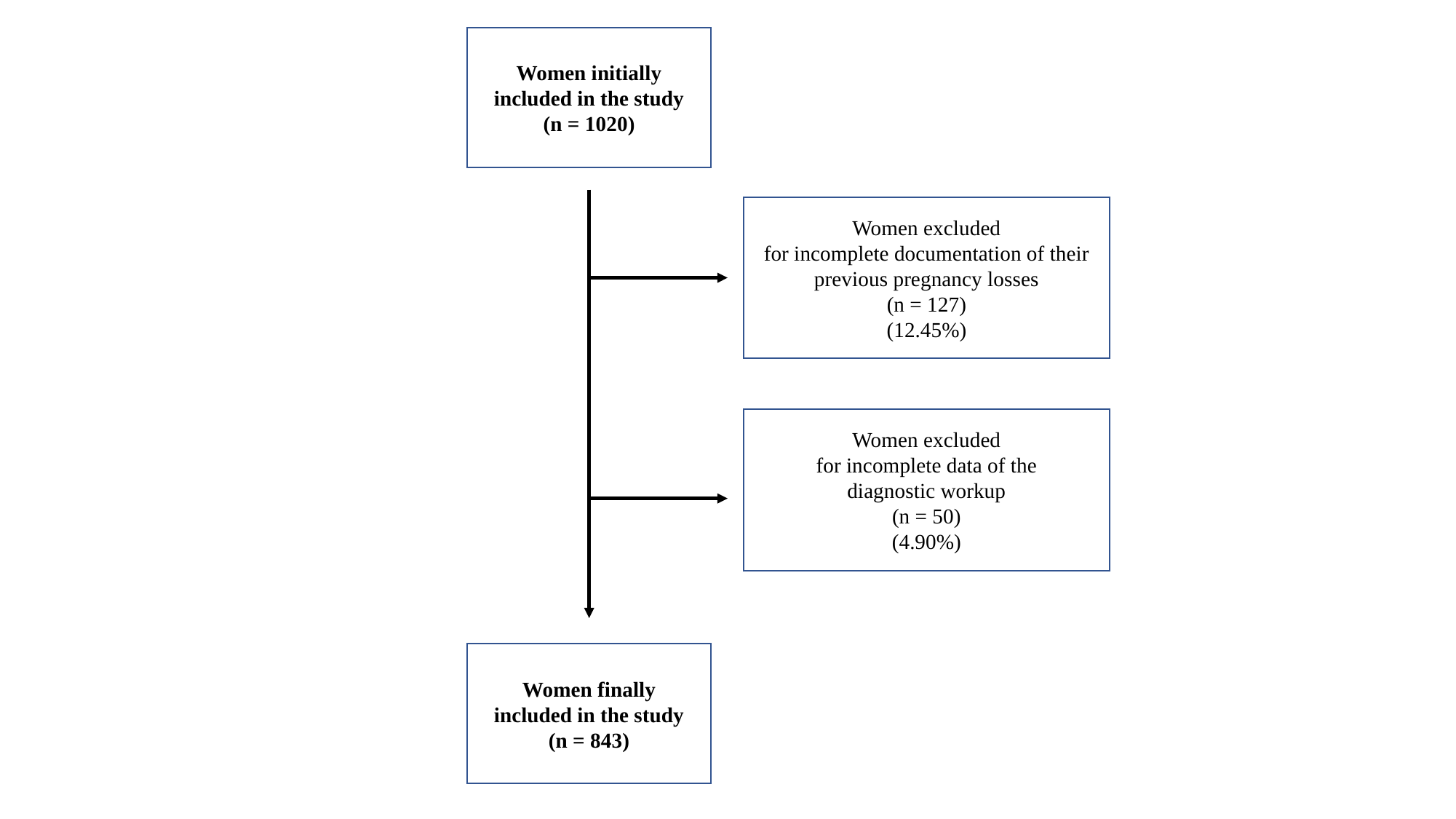

Women initially
included in the study
(n = 1020)
Women excluded
for incomplete documentation of their
previous pregnancy losses
(n = 127)
(12.45%)
Women excluded
for incomplete data of the
diagnostic workup
(n = 50)
(4.90%)
Women finally
included in the study
(n = 843)
