# Supplementary material for: Diagnostic factors for recurrent pregnancy loss: an expanded workup
Source: Arch Gynecol Obstet. 2023 Mar 25;308(1):127–42. doi: 10.1007/s00404-023-07001-z (PMC10191960; doi:10.1007/s00404-023-07001-z)
Supplement: Supplementary file 2 — (DOCX 12 KB) [file 404_2023_7001_MOESM2_ESM.docx]

**Supplemental Fig.1:**

Flow chart showing the patient selection workflow
