# Supplementary material for: Diagnostic factors for recurrent pregnancy loss: an expanded workup
Source: Arch Gynecol Obstet. 2023 Mar 25;308(1):127–42. doi: 10.1007/s00404-023-07001-z (PMC10191960; doi:10.1007/s00404-023-07001-z)
Supplement: Supplementary file 3 — (PDF 294 KB) [file 404_2023_7001_MOESM3_ESM.pdf]

PTV
FONDAZIONE PTV
POLICLINICO TOR VERGATA

Comitato Etico Indipendente
(D.M. 8 febbraio 2013)

PROTOCOLLO DI STUDIO
REGISTRO SPERIMENTAZIONI 42/19

Roma, 26 marzo 2019

Chiar.mo
Prof. Carlo Ticconi
U.O.C. Ginecologia
Fondazione PTV
Policlinico Tor Vergata
SEDE

RIUNIONE DEL 13 MARZO 2019

MEMBRI PRESENTI:

Prof. Massimo ANDREONI
Prof.ssa Maria Luisa BARBACCIA
Dott. Alfonso BELLIA
Prof.ssa Livia BIANCONE
Dott. Virgilio CALZINI
Dott.ssa Elena CAMPIONE
Dott.ssa Maria Grazia CELESTE
Prof. Carlo CHIARAMONTE
Dott.ssa Patrizia DANIELI
Prof. Claudio FRANCHINI
Prof.ssa Maria Luisa MANCA BITTI
Prof.ssa Maria Grazia MARCIANI
Dott.ssa Marcella MARLETTA
Prof. Saverio POTENZA
Prof. Mario ROSELLI
Prof.ssa Federica SANGIUOLO
Prof. Claudio SARTEA
Prof. Paolo SBRACCIA
Dott. Alessandro SILI
Prof. Umberto TARANTINO

MEMBRI ASSENTI GIUSTIFICATI:

Prof. William ARCESE
Dott. M. Andrea MANTO
Dott. Ercole VELLONE

Il Comitato Etico, si è riunito in data **13 marzo 2019** per esprimere il proprio Parere etico motivato sul **Protocollo di Studio** "I Fattori di Rischio dell'Aborto Spontanea Ricorrente".
Sperimentatore: Prof. Ticconi Carlo, U.O.C. Ginecologia
Promotore (no profit): Fondazione PTV Policlinico Tor Vergata; Università degli Studi di Roma Tor Vergata

ESAMINATA

la documentazione presentata e in particolare:

- Modulo di richiesta valutazione Studio (atti prot. 0006080/2019 del 06/03/2019);
- Dichiarazione sulla Natura no profit dello Studio;
- Protocollo di Studio (versione 05/03/2019).

RILEVA CHE

il Protocollo presentato:

- **è** giustificato scientificamente ed eticamente quanto al razionale
- **è** giustificato quanto al disegno e al piano statistico per l'analisi dei dati
- **è** giustificato quanto ai soggetti in studio
- **è** conforme alle disposizioni di legge ed alle conseguenti raccomandazioni di questo Comitato Etico Indipendente, in materia di rispetto della privacy (ai sensi del D.Lgs.196 del 30.06.2003; GDPR 679/2016)
- **è** giustificato quanto alla qualificazione del ricercatore e/o delle strutture

ESPRIME PARERE FAVOREVOLE

Il Parere è stato espresso dal Comitato all'unanimità dei votanti.

Si dichiara che questo Comitato è organizzato ed opera nel rispetto delle norme di Buona Pratica Clinica (GCP-ICH) e secondo la normativa vigente sulle Sperimentazioni Cliniche e sull'istituzione e funzione dei Comitati Etici.

Il Presidente del Comitato Etico
Prof.ssa Maria Grazia Marciani

Presidente

Prof.ssa M. Grazia MARCIANI<br>(componente esterno)	***"Clinico"***<br>Docens Turris Vergatae Neurologia<br>Università degli Studi di Roma "Tor Vergata"
Prof. Massimo ANDREONI<br>(componente interno)	***"Clinico"***<br>Ordinario di Malattie Infettive<br>Università degli Studi di Roma di "Tor Vergata"<br>Direttore U.O.C. Servizio di Malattie Infettive e Day Hospital<br>Fondazione PTV Policlinico Tor Vergata
Dott.ssa Lucy ANTONINI<br>(componente interno)	***"Farmacista del SSR"***<br>Farmacista afferente alla U.O.C. Farmacia Clinica<br>Fondazione PTV Policlinico Tor Vergata
Prof. William ARCESE<br>(componente interno)	***"Clinico"***<br>Ordinatorio di Ematologia<br>Direttore U.O.C. Trapianto Cellule staminali<br>Fondazione PTV Policlinico Tor Vergata
Prof.ssa Maria Luisa BARBACCIA<br>(componente esterno)	***"Farmacologo"***<br>Ordinario di Farmacologia<br>Dipartimento di Medicina dei sistemi<br>Università degli Studi di Roma "Tor Vergata"
Dott. Alfonso BELLIA<br>(componente interno)	***"Clinico"***<br>Ricercatore Università degli Studi di Roma Tor Vergata<br>U.O.C. Endocrinologia, Diabetologia e Malattie del metabolismo<br>Fondazione PTV Policlinico Tor Vergata
Prof.ssa Livia BIANCONE<br>(componente interno)	***"Clinico"***<br>Associato di Gastroenterologia<br>Università degli studi di Roma Tor Vergata<br>U.O.C. Gastroenterologia ed Endoscopia digestiva<br>Fondazione PTV Policlinico Tor Vergata
Dott.ssa Elena CAMPIONE<br>(componente interno)	***"Clinico"***<br>U.O.C. Dermatologia<br>Fondazione PTV Policlinico Tor Vergata

Dott. Virgilio CALZINI
(componente esterno)

"Medico di medicina generale territoriale"
Medico Esperto in Medicina Generale
associato FIMMG

Dott.ssa Maria Grazia CELESTE
(componente interno)

"Farmacista del SSR"
Direttore di U.O.C. Farmacia aziendale
Fondazione PTV Policlinico Tor Vergata

Prof. Carlo CHIARAMONTE
(componente esterno)

"Biostatistico"
Biostatistica e matematica attuariale
Docente a contratto di Lauree triennali della
Facoltà di Medicina e Chirurgia
Università degli Studi di Roma "Tor Vergata"

Dr.ssa Patrizia DANIELI
(componente esterno)

"Rappresentante del volontariato per l'assistenza e/o dell'associazionismo di tutela dei pazienti"
Presidente Associazione Nazionale
Noi negli Altri (A.N.N.A.)

Prof. Claudio FRANCHINI
(componente esterno)

"Esperto in materia giuridica e assicurativa"
Ordinario di Diritto amministrativo
Dipartimento di Giurisprudenza
Università degli Studi di Roma "Tor Vergata"

Prof. Andrea MAGRINI
(componente interno)

"Direttore sanitario o suo sostituto permanente"
Direttore Sanitario
Fondazione PTV Policlinico Tor Vergata

Prof.ssa Maria Luisa MANCA BITTI
(componente interno)

"Pediatria"
Aggregato di Pediatria Generale e Specialistica
Università degli Studi di Roma "Tor Vergata"
U.O.S.D. Pediatria e Gastroenterologia pediatrica
Fondazione PTV Policlinico Tor Vergata

Dott. M. Andrea MANTO
(componente esterno)

"Esperto di Bioetica"
Direttore Centro per la Pastorale Sanitaria
Diocesi di Roma

Dott.ssa Marcella MARLETTA
(componente esterno)

"Esperto in dispositivi medici"
Direttore Generale - Direzione Generale dei Dispositivi Medici del Servizio farmaceutico e della sicurezza delle cure, Ministero della Salute

Prof. Saverio POTENZA
(componente esterno)

"Medico legale"
Aggregato di Medicina Legale
Dipartimento di Medicina sperimentale e Chirurgia
Università degli Studi di Roma "Tor Vergata"

Prof. Francesco ROMEO
(componente interno, a chiamata)

"Esperto clinico del settore, in relazione allo studio di nuove procedure tecniche diagnostiche e terapeutiche invasive e semi invasive"
Ordinario di Cardiologia
Università degli Studi di Roma "Tor Vergata"
Direttore U.O.C. Cardiologia e Cardiologia interventistica
Fondazione PTV Policlinico Tor Vergata

Prof. Mario ROSELLI
(componente interno)

"Clinico"
Associato di Oncologia
Università degli Studi di Roma "Tor Vergata"
Dirigente responsabile U.O.S.D: Oncologia medica
Fondazione PTV Policlinico Tor Vergata

Prof.ssa Federica SANGIUOLO
(componente interno)

"Esperto di Genetica"
Associato di Genetica Medica
Università degli Studi di Roma "Tor Vergata"
U.O.C. Genetica Medica
Fondazione PTV Policlinico Tor Vergata

Prof. Claudio SARTEA
(componente esterno)

"Esperto di Bioetica"
Aggregato di Filosofia del Diritto
Dipartimento di Giurisprudenza
Università degli Studi di Roma "Tor Vergata"

Prof. Paolo SBRACCIA
(componente interno,a chiamata)

"***Clinico Esperto in nutrizione, in presenza di studi su prodotti alimentari"***
Ordinario di Medicina Interna
Direttore f.f. U.O.C. Medicina,
P.A. Centro di Eccellenza Cura dell'Obesità e Scienze Dietetiche
Fondazione PTV Policlinico Tor Vergata

Dott. Alessandro SILI
(componente interno)

"Rappresentante dell'area delle professioni sanitarie"
Coordinatore infermieristico
Responsabile U.O.C. Direzione Infermieristica e delle Professioni Sanitarie
Fondazione PTV Policlinico Tor Vergata

Prof. Umberto TARANTINO
(componente interno, a chiamata)

"Esperto qualificato, in relazione all'area medico chirurgica oggetto dell'indagine con il dispositivo medico in studio"
Ordinario di Malattie Apparato Locomotore
Università degli Studi di Roma "Tor Vergata"
Direttore U.O.C Ortopedia e Traumatologia b
Fondazione PTV Policlinico Tor Vergata

Dott. Ercole VELLONE
(componente esterno)

"Rappresentante dell'area professioni sanitarie"
Ricercatore di Scienze infermieristiche generali cliniche e pediatriche
Università degli Studi di Roma "Tor Vergata"
