# Supplementary material for: Diagnostic factors for recurrent pregnancy loss: an expanded workup
Source: Arch Gynecol Obstet. 2023 Mar 25;308(1):127–42. doi: 10.1007/s00404-023-07001-z (PMC10191960; doi:10.1007/s00404-023-07001-z)
Supplement: Supplementary file 4 — (DOCX 15 KB) [file 404_2023_7001_MOESM4_ESM.docx]

**Supplemental Tab. 1**

**Number of abnormalities in the diagnostic factors found in study women with 2 and > 3 pregnancy losses (PLs)**

| **Number of abnormalities in the diagnostic factors** | **Women with 2 PLs [n = 421]**  **(%)** | **Women with > 3 PLs [n = 422] (%)** | **OR (95% C.I.) for >3 vs 2 losses** | **P** |
| --- | --- | --- | --- | --- |
| No abnormalities | 23 (5,46%) | 0 | O.R.= 49.83 (3.01-832.16) | **0.0063** |
| 1 | 89 (21,14%) | 72 (17,06%) | O.R. = 1.30 (0.92-1.84) | 0.132, NS |
| 2 | 119 (28,26%) | 106 (25,11%) | O.R. = 1.17 (0.86-1.59) | 0.30, NS |
| 3 | 106 (25,17%) | 132 (31,27%) | O.R. = 0.73 (0.54-0.99) | **0.049** |
| 4 | 67 (15,91%) | 84 (19,90%) | O.R. = 0.76 (0.53-1.08) | 0.13, NS |
| 5 | 17 (4,03%) | 24 (5,68%) | O.R. = 0.69 (0.36-1.31) | 0.26, NS |
| 6 | 0 | 4 (0,94%) | O.R. = 0.11 (0.005-2.055) | 0.13, NS |

NS: not significant

Chi-square: 35.49, P = 0.0000034
