# Supplementary material for: Diagnostic factors for recurrent pregnancy loss: an expanded workup
Source: Arch Gynecol Obstet. 2023 Mar 25;308(1):127–42. doi: 10.1007/s00404-023-07001-z (PMC10191960; doi:10.1007/s00404-023-07001-z)
Supplement: Supplementary file 5 — (DOCX 15 KB) [file 404_2023_7001_MOESM5_ESM.docx]

**Supplemental Tab. 2**

**Number of abnormalities in the diagnostic factors found in study women according to the type of RPL – Primary or secondary**

| **Number of abnormalities in the diagnostic factors** | **Women with Primary RPL**  **[n = 532]**  **(%)** | **Women with Secondary RPL**  **[n = 311]**  **(%)** | **O.R. (95% C.I.) for Primary vs**  **Secondary RPL** | **P** |
| --- | --- | --- | --- | --- |
| No abnormalities | 12 (2.25%) | 11 (3.53%) | O.R. = 0.62 (0.27-1.44) | P = 0.27, NS |
| 1 | 90 (16.91%) | 71 (22.82%) | O.R. = 0.68 (0.48-0.97) | **P = 0.03** |
| 2 | 148 (27.81%) | 77 (24.75%) | O.R. = 1.17 (0.85-1.61) | P = 0.33, NS |
| 3 | 151 (28.38%) | 87 (27.97%) | O.R. = 0.89 (0.74-1.39) | P = 0.89, NS |
| 4 | 97 (18.23%) | 54 (17.36%) | O.R. = 1.06 (0.73-1.53) | P = 0.75, NS |
| 5 | 31 (5.82%) | 10 (3.21%) | O.R. = 1.86 (0.90-3.85) | P = 0.09, NS |
| 6 | 3 (0.56%) | 1 (0.32%) | O.R. = 1.75 (0.18-16.97) | P = 0.62, NS |

NS = not significant

Chi-square: 8.55, P = 0.07, NS
